# Active versus sham DLPFC-NAc rTMS for depressed adolescents with anhedonia using resting-state functional magnetic resonance imaging (fMRI): a study protocol for a randomized placebo-controlled trial

**DOI:** 10.1186/s13063-023-07814-y

**Published:** 2024-01-13

**Authors:** Runxin Lv, Min Cai, Nailong Tang, Yifan Shi, Yuyu Zhang, Nian Liu, Tianle Han, Yaochi Zhang, Huaning Wang

**Affiliations:** 1 Department of Psychiatry of Xijing Hospital of Air Force Medical University, 127 Changle West Road, Xi’an, Shaanxi Province China; 2 Department of Psychiatry, 907 Hospital, No. 99 Binjiang North Road, Yanping District, Nanping City, Fujian Province China

**Keywords:** Transcranial magnetic stimulation, Anhedonia, Depressive disorder, Major, Neuromodulation, Nucleus accumbens, Randomized controlled trial

## Abstract

**Background:**

Anhedonia, which is defined as the inability to feel pleasure, is considered a core symptom of major depressive disorder (MDD). It can lead to several adverse outcomes in adolescents, including heightened disease severity, resistance to antidepressants, recurrence of MDD, and even suicide. Specifically, patients who suffer from anhedonia may exhibit a limited response to selective serotonin reuptake inhibitors (SSRIs) and cognitive behavioral therapy (CBT). Previous researches have revealed a link between anhedonia and abnormalities within the reward circuitry, making the nucleus accumbens (NAc) a potential target for treatment. However, since the NAc is deep within the brain, repetitive transcranial magnetic stimulation (rTMS) has the potential to modulate this specific region. Recent advances have enabled treatment technology to precisely target the left dorsolateral prefrontal cortex (DLPFC) and modify the functional connectivity (FC) between DLPFC and NAc in adolescent patients with anhedonia. Therefore, we plan to conduct a study to explore the safety and effectiveness of using resting-state functional connectivity magnetic resonance imaging (fcMRI)-guided rTMS to alleviate anhedonia in adolescents diagnosed with MDD.

**Methods:**

The aim of this article is to provide a study protocol for a parallel-group randomized, double-blind, placebo-controlled experiment. The study will involve 88 participants who will be randomly assigned to receive either active rTMS or sham rTMS. The primary object is to measure the percentage change in the severity of anhedonia, using the Snaith-Hamilton Pleasure Scale (SHAPS). The assessment will be conducted from the baseline to 8-week post-treatment period. The secondary outcome includes encompassing fMRI measurements, scores on the 17-item Hamilton Rating Scale for Depression (HAMD-17), the Montgomery Asberg Depression Rating Scale (MADRS), the Chinese Version of Temporal Experience of Pleasure Scale (CV-TEPS), and the Chinese Version of Beck Scale for Suicide Ideation (BSI-CV). The Clinical Global Impression (CGI) scores will also be taken into account, and adverse events will be monitored. These evaluations will be conducted at baseline, as well as at 1, 2, 4, and 8 weeks.

**Discussion:**

If the hypothesis of the current study is confirmed, (fcMRI)-guided rTMS could be a powerful tool to alleviate the core symptoms of MDD and provide essential data to explore the mechanism of anhedonia.

**Trial registration:**

ClinicalTrials.gov NCT05544071. Registered on 16 September 2022.

**Supplementary Information:**

The online version contains supplementary material available at 10.1186/s13063-023-07814-y.

## Introduction

### Background and rationale

Major depressive disorder (MDD) is a common mental disorder among adolescents worldwide [1], By the end of puberty, up to 20% of adolescents may have experienced it [2], leading to a significant burden in both low-income and high-income countries [3, 4]. The COVID-19 pandemic has further increased the prevalence of anxiety and depression, with youth being one of the most affected groups [5]. Depression in adolescents is closely associated with suicide and other adverse outcomes, such as recurrent MDD in adulthood, impaired social function, and physical illness [4, 6, 7].

Anhedonia, the inability to experience pleasure, is a key marker of depression vulnerability [8] and significantly contributes to the severity of depression. It predicts reduced treatment efficacy and an elevated risk of relapse and suicidality [9–11]. Despite access to empirically supported treatments like cognitive-behavioral therapy (CBT), selective serotonin reuptake inhibitors (SSRIs), or their combination, many adolescents with depression exhibit treatment resistance [12]. Anhedonia, therefore, plays a detrimental role in the onset, management, and prognosis of MDD in adolescents. Yet, the therapeutic outcomes for addressing anhedonia remain far from optimal.

Previous researches have underscored a connection between anhedonia and irregularities within the reward circuitry. Individuals with MDD [13, 14], who frequently experience anhedonia [15] show weaker neurobiological responses to anticipating rewards when compared to healthy controls. This is most pronounced in the nucleus accumbens (NAc), which plays a crucial role in reward processing [16]. A comprehensive longitudinal study involving adolescents has found that a diminished response in the ventral striatum during reward anticipation can presage the onset of anhedonia two years later in adolescents who were initially healthy. This predictive relationship is distinct from the one involving low mood without anhedonia [17, 18]. This indicates that the ventral striatum, particularly the NAc, could be a biomarker and potential target for anhedonia. However, conventional noninvasive techniques for treating the NAc can be difficult due to its deep-seated position in the brain. Repetitive transcranial magnetic stimulation (rTMS) may offer a solution by indirectly influencing the NAc through functional connectivity (FC) with the brain’s superficial cortex [19]. Studies employing rTMS to target the most positively correlated FC between the dorsolateral prefrontal cortex (DLPFC) and the NAc have shown significant amelioration in anhedonia in adults subjects [20]. However, effective intervention targets for anhedonia in adolescent patients, a crucial period for managing MDD, remain elusive. Therefore, testing this hypothesis specifically within this demographic is paramount and forms the basis of our study.

RTMS is a safe and noninvasive neuromodulation technology that has received FDA approval [21]. By intensifying synaptic connections and modifying FC and activity patterns in distant regions of the same brain region, high-frequency rTMS has demonstrated the potential to treat mental disorders, particularly MDD [22, 23], through a novel approach called individual target-transcranial magnetic stimulation (IT-TMS) [24–26]. It is believed to be the mechanism by which rTMS addresses anhedonia, a hallmark symptom of MDD, involves enhancing the FC between the left DLPFC and the NAc using IT-TMS [19].

We are planning to conduct a randomized controlled trial (RCT) to evaluate the efficacy of rTMS in managing anhedonia, which is a core symptom of MDD, in adolescents. The specific target areas for this study will be the DLPFC-NAc. We will use an intelligent neural navigation system to generate personalized targets based on resting-state fcMRI analysis, which will involve identifying the left DLPFC region that has the highest functional connectivity with the NAc in each participant. Our research aims to determine the preliminary efficacy, safety, and tolerability of the rTMS protocol using fcMRI-guided targeting. We hypothesized that adolescents who suffer from MDD and anhedonia will have altered neural functional patterns. We anticipate that these patterns will normalize after exposure to stimuli. Additionally, we aimed to explore changes in FC and neuropsychological functioning that are associated with anhedonia and MDD. By conducting this study, we hope to demonstrate that our hypothesis has the potential to improve the treatment of anhedonia.

### The explanation for the choice of comparators

All patients will receive pharmacological treatments and be randomly assigned to either the active rTMS group or the sham rTMS group with a 1:1 distribution. They will then undergo consecutive intervention treatment for 15 days.

### Objective

This study aims to evaluate the efficacy and safety of targeted rTMS in managing anhedonia in adolescents with MDD by precisely targeting DLPFC-NAc FC.

### Trial design

**Fig. 1:**
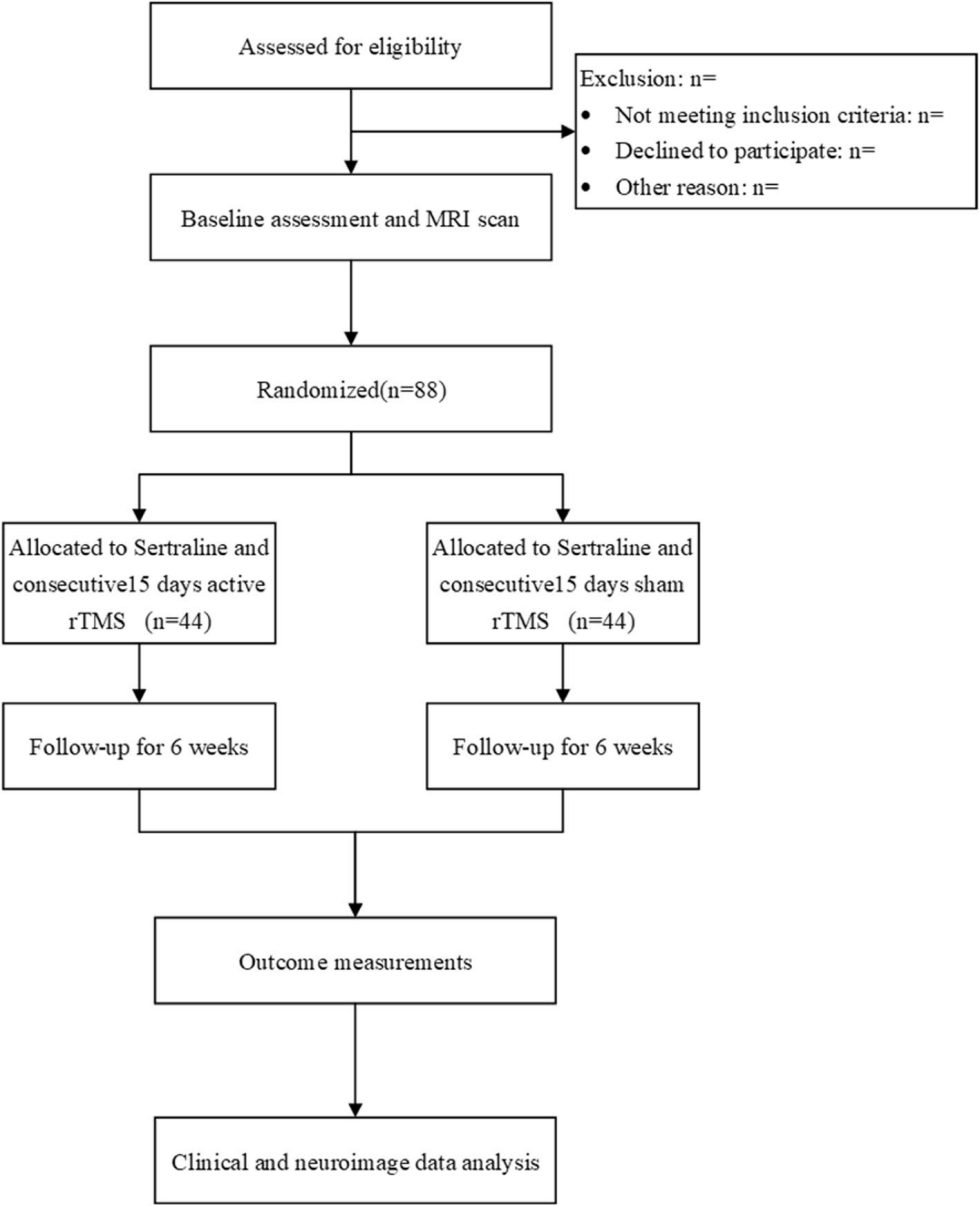
Flow chart to interpret the format of the trial

This study is a double-blind, placebo-controlled, parallel-group, randomized superiority trial sponsored by the Department of Psychiatry at Xijing Hospital. The Ethics Committee of Xijing Hospital in Shaanxi Province, China, has granted approval for this study, which is also registered with ClinicalTrials.gov (reference number: NCT05544071). The study’s design is depicted in Fig. 1. Furthermore, we will follow the guidelines set by SPIRIT for reporting throughout the trial [27] (Additional file 2).

## Methods: participants, interventions, and outcomes

### Study setting

This study will be conducted at Xijing Hospital (Xian, China). It is an affiliated hospital of the Air Force Medical University and sees an average 12,000 outpatients per day. From February 2023 to December 2023, eligible adolescent MDD patients will be screened for participation in both outpatient and inpatient settings. A total of 88 patients will be recruited for trial.

### Eligibility criteria

Researchers will conduct eligibility screening based on inclusion and exclusion criteria. All participants must provide voluntary consent and sign an informed consent form after qualification is confirmed (Additional file 1).

#### Inclusion criteria

Participants are eligible for this study if they meet all of the following criteria.

1. Aged between 13 (inclusive) and 18 (exclusive) years.
2. Diagnosed with MDD and currently experiencing a major depressive episode, as per the Diagnostic and Statistical Manual of Mental Disorders, 5th edition.
3. Must secure a total score of 17 or higher on the Hamilton Rating Scale for Depression (HAMD-17) during both the screening and baseline visits (day − 7 and day 0).
4. As per academic requirements, participants should achieve a total score of 20 or more on the Snaith-Hamilton Pleasure Scale (SHAPS) during both the screening and baseline visits, which occur on day − 7 and day 0 respectively [28].
5. Good overall health status, as verified by medical records.
6. After gaining a comprehensive understanding of the rTMS treatment, participants must express their eagerness to actively participate in the therapy and be capable of providing informed consent.

#### Exclusion criteria

The study will exclude participants who meet any of the following criteria.

1. Current diagnosis of a substance use disorder, excluding nicotine and caffeine dependency.
2. Current diagnosis of any mental disorders other than dysthymic disorder, generalized anxiety disorder, social anxiety disorder, panic disorder, agoraphobia, or specific phobia. If these disorders are clinically unstable or have been the primary focus of treatment for the past 6 months or more, they should be excluded.
3. History of schizophrenia or schizoaffective disorders, or presence of psychotic symptoms during current or past episodes of depression.
4. Presence of any additional mental disorders, personality disorders, or intellectual disabilities that are clinically more significant than MDD at screening.
5. Clinically abnormality detected during screening that could potentially affect safety, interfere with study participation, or complicate the interpretation of study results.
6. Any current or past physical condition that, in the investigator’s judgment, could pose a risk to the participant or interfere with the interpretation of study results.
7. Participation in other clinical trials involving an investigational drug or device within the past month or during the study period.
8. Presence of electronic devices or metal implants in the skull.
9. History of epilepsy.
10. Previous history of cardiovascular disease or cardiac event.
11. Prior diagnosis of obsessive–compulsive disorder.
12. History of autism spectrum disorder.
13. Previously exposed to rTMS.
14. Use of any antidepressants within 2 weeks prior to the screening phase.
15. Any other circumstances deemed by the investigators as inappropriate for the study.

### Interventions: description

The primary interventions in this study encompass both rTMS and pharmacological treatments.

#### rTMS

The study will employ a Black Dolphin TMS Robot (Spirit Dolphin, SLD-YXRJ-V1.0, Solide company, Xi'an, China) equipped with a figure-of-eight-shaped coil to apply rTMS. A personalized 3D facial tracer will be created based on participants’ MRI data, enabling the robotic arm to position the rTMS probe accurately onto the target stimulation area during treatment. Before initiating treatment, the rest motor threshold (rMT) will be determined. The rMT is the minimum intensity needed to generate 5 motor-evoked potential responses out of 10 attempts.

In the active rTMS group, we will administer 50 sessions of stimulation. Each session will include 6-s trains operating at 10 Hz, with a 30-s interval between trains. The stimulation will specifically target left DLPFC, which has the strongest functional correlation with NAc. Each session will deliver 3000 pulses over 15 consecutive days, resulting in a total of 45,000 pulses. The stimulation will be administered at an intensity of 120% of the rMT.

The procedure will be identical in the sham group, except that the coil will be positioned at a 90° angle to the scalp surface, thereby mimicking the procedure without delivering active stimulation.

#### Medication

During the rTMS treatment, participants will receive sertraline hydrochloride tablets, as the China National Medical Products Administration has approved sertraline for treating MDD in adolescents. Participants will begin taking sertraline hydrochloride tablets post-screening. The initial dosage will be 25 mg/day for the first 3 days, 50 mg/day for the subsequent 4 days, further increased to 100 mg /day after 1 week, assuming no adverse events that limit dosage. The dose can be flexibly adjusted between 100 and 150 mg/day until an optimal clinical response. Subsequently, participants will be required to maintain a stable antidepressant medication regimen. Concomitant medication for insomnia, such as benzodiazepines or nonbenzodiazepine hypnotics, will be permitted in this study. Any other medications taken will be reported to the principal investigator throughout the study period. Furthermore, the case report form (CRF) will meticulously document all administered medications.

#### MRI

The study will utilize a 3.0-T UNITED 770 scanner for MRI data acquisition before and after treatment. Subjects will lie quietly on the bed during the procedure, using cotton pads to minimize head motion and earplugs to block out extraneous noise. The parameters for 3D-T1-weighted structural imaging will be set as follows:

- Number of slices = 192
- Repetition time = 7.24 ms
- Echo time = 3.10 ms
- Slice thickness = 1.0 mm
- Matrix size = 512 × 512
- Field of view = 256 × 256 mm^2^
- Flip angle = 10°

The acquisition parameters will be set as follows:

- Number of slices = 35
- Repetition time = 2000 ms
- Echo time = 30 ms
- Slice thickness = 4 mm
- Matrix size = 64 × 64
- Field of view = 224 × 224 mm^2^
- Flip angle = 90°

These settings will ensure high-resolution imaging necessary for precise analysis of structural brain changes.

### Interventions: modifications

There is no intervention modification plan except for adjusting drug dosage due to changes in symptoms.

### Interventions: adherence

Before intervention implementation, the operator will provide participants with a detailed introduction to the treatment to dispel patient concerns, including its nature, expected effects, and possible adverse reactions. During treatment, they will also be reminded of follow-up treatment time. The purpose of these measures is to improve adherence to intervention protocols.

### Interventions: concomitant care

Concomitant medication for insomnia, such as benzodiazepines or nonbenzodiazepine hypnotics, will be permitted in this study. Any other medications taken will be reported to the principal investigator and meticulously documented in the case report form (CRF) throughout the study period.

### Outcome

#### Primary outcome

The primary outcome measure for this study will be the percentage difference in the SHAPS score between the initial (baseline) assessment and the 8-week follow-up assessment. The SHAPS is a widely adopted 14-item self-assessment questionnaire utilized to gauge the severity of anhedonia symptoms in individuals diagnosed with mood disorders. The scale ranges from 14 to 56, with higher scores indicating more severe anhedonia symptoms.

#### Secondary outcome

Secondary outcomes of this research will include the following:

1. Seventeen-item Hamilton Depression Rating Scale (HAMD-17): Percent decrease in the HAMD-17 and the Montgomery Asberg Depression Rating Scale (MADRS) to assess remission and recovery from depression. The HAMD is the most commonly used clinician-administered scale for evaluating depression [29]. This study will use the 17-item, where a higher score denotes more severe symptoms.
2. Montgomery Asberg Depression Rating Scale (MADRS): This examiner-rated scale can be used to evaluate the depressive symptoms of MDD in conjunction with HAMD-17 [30]. The scale ranges from 0 to 60, with a higher score indicating more significant depressive symptomology.
3. The Chinese Version of Temporal Experience of Pleasure Scale (CV-TEPS): The TEPS includes 20 items, 11 measuring anticipatory anhedonia and 9 evaluating consummatory anhedonia [31, 32]. The Chinese version (CV-TEPS) has been confirmed as a reliable and valid instrument for assessing anhedonia in Chinese students [32]. The scale ranges from 20 to 120, with a lower score representing more significant anhedonia symptomology.
4. The Chinese Version of Beck Scale for Suicide Ideation (BSI-CV). The BSI-CV is a 19-item questionnaire used to reliably and validly measure the severity of suicidal ideation [33, 34]. The scale includes two subscales: current suicidal ideation and past suicidal ideation (Suicidal ideation at one’s worst point). The scale ranges from 0 to 38, with higher scores indicating more severe suicidal ideation in the last week or at the most serious point.
5. The Insomnia Severity Index (ISI): The ISI questionnaire has good reliability and validity for estimating insomnia severity. It includes 7 items: the severity of insomnia symptoms, satisfaction with sleep patterns, the effects of insomnia on daytime function, the effects of insomnia on subjects’ quality of life, and the degree of worry or distress caused by insomnia [35, 36]. The scale ranges from 0 to 28, with a higher score illustrating more significant insomnia symptomology.
6. The Clinical Global Impression (CGI): The CGI scale is designed to assess clinical efficacy and includes three parts: severity of illness, global improvement, and effect index [37]. A higher score suggests a more severe disease and poorer efficacy.
7. FC: Functional connectivity refers to the extent to which two brain regions are simultaneously activated and communicating with each other [38]. It is recorded using fMRI techniques, such as the blood oxygen level-dependent (BOLD) signal.
8. THINC-it: THINC-it is a cognitive screening tool comprised of objective and subjective measures of cognitive function with high levels of reliability and stability [39].

### Participant timeline

**Fig. 2:**
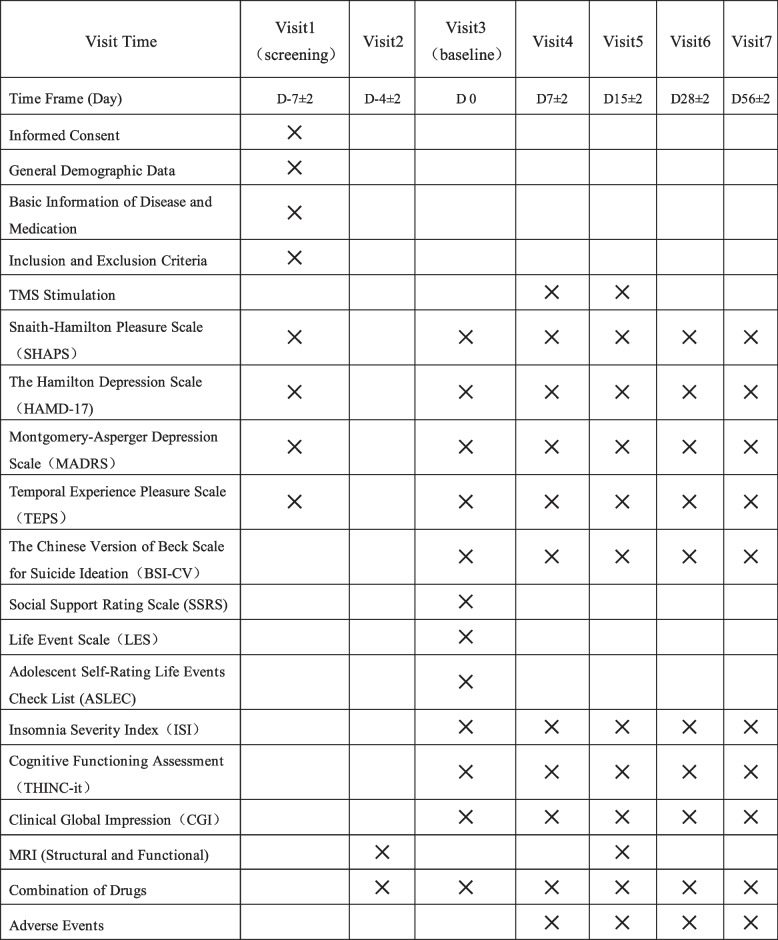
Case report form (CRF)

This study will be divided into a 7-day screening period, a 15-day stimulation period, and a 6-week follow-up period. After qualification confirmation, the subjects will sign an informed consent form, undergo MRI scans, and begin sertraline titration. Subsequently, they will be randomly assigned to one of two groups for a 15-day stimulation period with a 1:1 ratio. All patients will undergo a second MRI scan after the stimulation period. Finally, there will be a 6-week follow-up period. We will conduct clinical scale evaluation and cognitive testing at baseline (D0), 7 and 15 days of rTMS treatment (D7&D15), and 2 and 6 weeks after the end of the stimulation period (D28&D56). We will track the number of participants excluded, refused consent, or withdrawn from the study and their reasons. The timeline is shown in Fig. 2.

### Sample size

The objective of this study is to investigate the efficacy and underlying mechanism of rTMS, explicitly targeting the DLPFC and NAc in adolescents suffering from MDD and anhedonia. Based on previous studies [40], we anticipate the SHAPS score to be 29.59 ± 7.677 in the active treatment group compared to 34.82 ± 7.283 in the sham treatment group. A power analysis (with a power of 0.8, *α* of 0.05, and a two-sided test) estimates that a minimum of 34 patients per group will be required to detect a significant difference. Taking into account a projected dropout rate of 20% and blocked randomization, the plan is to recruit a total of 88 patients. As for the fMRI studies, earlier studies suggest a sample size between 15 and 30 patients suffices to validate the hypothesis [41]. Therefore, this study will proceed with the computed maximum sample size of 88.

### Recruitment

This study will recruit participants who fulfill our criteria from both the outpatient and inpatient departments of Xijing Hospital, utilizing recruitment advertisements for outreach.

## Methods: assignment of interventions

### Allocation: sequence generation

The study will adopt a blocked randomization approach. The random allocation sequence will be generated using statistical analysis software (version 9.4, SAS Institute Inc., Cary, NC, USA). The purpose of generating sequence numbers is to divide participants evenly and randomly into two groups.

### Allocation: concealment mechanism

Computer-assisted generation of opaque envelopes will be marked with sequence numbers externally and containing a concealed randomization list internally. Upon confirmation of each patient’s eligibility, researchers will open the envelope and inform operators responsible for implementing treatment of the grouping results.

### Allocation: implementation

The unblinded research members will create the allocation sequence and assign participants to either the active or sham treatment groups. RTMS is performed by trained operators subsequently.

### Blinding

The treatment assignments will be hidden from participants, clinical evaluators, and statistical analysts. While aware of participant allocation, the unblinded research members and experienced rTMS operators will be deliberately excluded from the evaluations and data analysis processes.

### Blinding: emergency unblinding

They are barring exceptional circumstances such as serious adverse events or other emergent situations necessitating the breaking of the blinding protocol.

## Methods: data collection, management, and analysis

### Data collection plan

A paper case report form (CRF) will record all experimental processes and results. After each participant completes the research process, two researchers will independently input the original paper records into an Excel sheet to verify the completeness and accuracy of the paper records and data entry with each other. MRI scan will be implemented at the Yunying Medical Imaging Diagnosis Center (Xian, China).

### Data collection plan: retention

When signing the informed consent form, researchers will provide participants with a detailed introduction to the trial situation, including the purpose, process, requirements, and disease-related information, including etiology, treatment methods, and outcomes. During the study process, they will also be reminded to complete subsequent actions. The purpose of these measures is to improve subject compliance. Subjects can request withdrawal for any reason, while researchers will collect as much outcome data on this group of patients as possible.

### Data management

Clinical data will be accurately, comprehensively, and promptly recorded on CRF as soon as they are acquired. Each participant will be assigned a unique identifier, replacing the need for personal names in the data collection process. After each participant completes the research, two researchers will independently input the original paper records into an Excel sheet to verify the completeness and accuracy of the paper records and data entry with each other. This database will allow the application for the corresponding author to access the final trial dataset. Data will be directly imported into IBM SPSS (version 26.0) to facilitate the statistical analysis.

### Statistics: outcomes

Statistical analyses will be conducted on clinical data using IBM SPSS version 26 (Armonk, NY). Current research results are expected to be consistent with the two-factor (active and sham rTMS) and multilevel (baseline and all follow-up time points) analysis of variance of repeated measurement data. We will evaluate the normality of continuous data through the Shapiro–Wilk test. To examine the assumption of sphericity, we will employ Mauchly’s test of sphericity. A two-sided test will be deemed statistically significant if the *P*-value is less than or equal to 0.05. Any missing data in a specific group will be substituted with the corresponding group’s mean value. Mean ± standard deviation or frequency (%) will be used to present sociodemographic information and clinical outcomes in both the active and sham treatment groups. The neuroimaging indexes will be analyzed by MATLAB R2013b and SPM12 software to explore the changes in FC from baseline to posttreatment. We used correlations between imaging data and questionnaire scores to identify predictors potentially affecting rTMS outcomes.

### Statistics: additional analyses

There are no additional analysis plans.

### Statistics: analysis population and missing data

Any missing data in a specific group will be substituted with the corresponding group’s mean value.

## Methods: monitoring

### Data monitoring: formal committee

A data monitoring committee (DMC) is crucial in maintaining researchers’ and clinicians’ blindness and ensuring the research study’s integrity. To address potential adverse reactions, an emergency epilepsy rescue team will be established under the purview of the DMC. Before enrollment, all researchers involved in the study will undergo training to understand the clinical manifestations and management processes related to seizures. In the event of any adverse reactions, these will be promptly reported to the DMC and relevant regulatory agencies. This procedure ensures the study participants’ safety and the study results’ validity.

### Data monitoring: interim analysis

There are no interim analysis plans.

### Harms

Possible adverse reactions associated with rTMS may include headache, fatigue, tingling, muscle twitching or soreness, syncope (fainting), mania, epilepsy, etc. All adverse reactions during the study will be meticulously recorded in the CRF. Each case will be evaluated for severity and possible causal relationships with the treatment. In the event of any adverse reactions, the information will be promptly reported to the DMC and relevant regulatory agencies. This ensures a high standard of safety and transparency throughout the study and allows for appropriate action if required.

### Auditing

The sponsoring agency will appoint independent supervisors to oversee the research supervision work of the clinical trials. The role of these inspectors is to ensure that researchers adhere strictly to the experimental protocol, relevant standard operating procedures, guiding principles, and regulatory requirements throughout the research process. The supervisors conduct monthly site visits to check the research progress. These visits will involve verification of original records, CRF, and other pertinent information. These checks aim to ensure the researcher’s compliance with the research protocol, procedures, and regulations and guarantee that the obtained research data is objective, truthful, and lawful. At the end of the study, the inspectors will be responsible for verifying and archiving all documents about the research conducted at the center.

## Discussion

This study is a randomized, double-blind, sham stimulation-controlled, parallel-group trial. Its goal is to investigate the effectiveness of rTMS in improving anhedonia in adolescent patients diagnosed with MDD. Previous researches have indicated that rTMS could be a promising therapeutic intervention for anhedonia [42]. However, the efficacy of fcMRI-guided rTMS in mitigating anhedonia in adolescent patients is yet to be explored. The brain’s neural network is a complex system that has been partially explored. Advances in fMRI have provided compelling evidence suggesting that individuals exhibit substantial activation in striatal regions (specifically, the NAc, caudate, and putamen) during reward processing. In contrast, individuals suffering from anhedonia often show diminished activity in these regions, regardless of whether they have MDD or not [43–46]. Randy et al. found that reduced reward-related activation in the NAc was linked to anhedonia in adolescents with first-episode depression [43]. Additionally, a recent study reported that activating the left ventral striatum, particularly the NAc, improves anhedonia-related symptoms [47]. This suggests that Nac could be a potential target for anhedonia [47–49]. As discussed earlier, the effectiveness of antidepressant medication and evidence-based psychotherapy remains unsatisfactory. However, rTMS shows promise as a precise intervention for the NAc. Due to the positively correlated functional connection between the left DLPFC and NAc [19], it is crucial to conduct additional clinical studies to clarify the effectiveness and potential neural mechanisms of fcMRI-guided rTMS through DLPFC-NAc FC for anhedonia in adolescent MDD patients. This clinical study will generate the necessary data to support these hypotheses.

We hypothesize a potential association between the efficacy of the intervention and the modulation of FC between the DLPFC and NAc. If this hypothesis is correct, the active treatment group should show higher modulation in the FC maps of reward-related regions than the sham treatment group. These results could provide insights into the potential mechanism of rTMS for this specific symptom among adolescents suffering from MDD.

## Trial status

The Ethics Committee of Xijing Hospital approved the study protocol in September 2022 (Protocol ID: KY20222165-F-1). The current version is version 4.0, dated 11 May 2023. This trial has been registered with the ClinicalTrials.gov database under the reference number NCT05544071. The trial commenced on 1 February 2023. Participant recruitment for the trial is ongoing. We have enrolled and randomly assigned 51 participants as of the latest update. We anticipate completing the recruitment phase by December 2023.

## Ethics and dissemination

### Research ethics approval

The study protocol has received approval from the Ethics Committee of Xijing Hospital in Shaanxi Province, China. and was registered at ClinicalTrials. Gov (URL: the Safety and Effectiveness of Precise rTMS Based on Neuroimaging in the Treatment of Adolescent Depression With Anhedoniadepression With Anhedonia—Full Text View—ClinicalTrials.gov reference number: NCT05544071). This study will be conducted in accordance with the ethical principles stated in the Declaration of Helsinki.

### Protocol amendments

Any significant modifications to the protocol will be promptly reported to the Ethics Committee of Xijing Hospital and updated on ClinicalTrials.gov.

### Consent or assent

Eligibility screening for adolescent MDD patients will be conducted in outpatient and inpatient at Xijing Hospital. The researchers will provide participants with a detailed introduction to the trial situation, including the purpose, process, and requirements after qualification confirmation. All participants will voluntarily participate in the study and sign an informed consent.

### Consent or assent: ancillary studies

There are no relevant plans.

### Confidentiality

After enrollment, each participant will be assigned a unique identifier, replacing the need for personal names in the data collection process. This unique identifier will be meaningful only to the research team, ensuring participant confidentiality. On completion of the study, the principal investigator will be responsible for securely storing and protecting these unique identifiers, in accordance with research guidelines. Any publications resulting from this study will not include any personally identifiable information, maintaining participant privacy at all times.

### Declaration of interests

The submitter declares that they have no competing interests.

### Data access

This database will allow the application for Huaning Wang to access the final trial dataset.

### Ancillary and post-trial care

If any harm related to this study occur, participants can receive free treatment provided by Xijing Hospital, which will compensate in accordance with relevant laws and regulations.

### Dissemination policy: trial results

The results of the study will be prepared for submission to international, peer-reviewed journals. This process involves assembling the data into a comprehensive manuscript that outlines the study’s methodology, findings, and implications.

### Dissemination policy: authorship

See the author’s contribution section below.

### Dissemination policy: reproducible research

The public can access the complete protocol through ClinicalTrials.gov website (NCT05544071), but it does not include the personal information of participants. This database will allow the reasonable application for corresponding author to access the final trial dataset.

### Supplementary Information

**Additional file 1.**

**Additional file 2.**

## Data Availability

All study data will be deposited at the Department of Psychiatry of Xijing Hospital. The datasets that have been analyzed during the current study will be available for future secondary analysis with the approval from the corresponding author.

## Abbreviations

MDD: Major depressive disorder
SSRI: Selective serotonin reuptake inhibitor
CBT: Cognitive behavioral therapy
NAc: Nucleus accumbens
rTMS: Repetitive transcranial magnetic stimulation
DLPFC: Dorsolateral prefrontal cortex
FC: Functional connectivity
MRI: Magnetic resonance imaging
SHAPS: Snaith-Hamilton pleasure scale
HAMD-17: Hamilton Rating Scale for Depression
MADRS: Montgomery Asberg Depression Rating Scale
CV-TEPS: Chinese Version of the Temporal Experience of Pleasure Scale
BSI-CV: Chinese Version of Beck Scale for Suicide Ideation
ISI: Insomnia Severity Index
CGI: Clinical Global Impression
IT-TMS: Individual target-transcranial magnetic stimulation
RCT: Randomized controlled trial
CRF: Case report form
RMT: Rest motor threshold
DMC: Data Monitoring Committee

## References

[1] WHO. Adolescent mental health 2020 [Available from: https://www.who.int/news-room/fact-sheets/detail/adolescent-mental-health. Accessed 24 Oct 2023.

[2] Thapar A, Collishaw S, Pine DS, Thapar AK (2012). Depression in adolescence. The Lancet.

[3] Lu J, Xu X, Huang Y, Li T, Ma C, Xu G (2021). Prevalence of depressive disorders and treatment in China: a cross-sectional epidemiological study. Lancet Psychiatry.

[4] Copeland WE, Alaie I, Jonsson U, Shanahan L (2021). Associations of Childhood and Adolescent Depression With Adult Psychiatric and Functional Outcomes. J Am Acad Child Adolesc Psychiatry.

[5] organization wh. COVID-19 pandemic triggers 25% increase in prevalence of anxiety and depression worldwide 2022 [Available from: https://www.who.int/news/item/02-03-2022-covid-19-pandemic-triggers-25-increase-in-prevalence-of-anxiety-and-depression-worldwide. Accessed 24 Oct 2023. PMC9998057 35414629

[6] Viswanathan M, Wallace IF, Cook Middleton J, Kennedy SM, McKeeman J, Hudson K (2022). Screening for depression and suicide risk in children and adolescents: updated evidence report and systematic review for the US Preventive Services Task Force. JAMA.

[7] Leone M, Kuja-Halkola R, Leval A, D’Onofrio BM, Larsson H, Lichtenstein P (2021). Association of youth depression with subsequent somatic diseases and premature death. JAMA Psychiat.

[8] Kovacs M, Lopez-Duran N (2010). Prodromal symptoms and atypical affectivity as predictors of major depression in juveniles: implications for prevention. J Child Psychol Psychiatry.

[9] Rubin DH (2012). Joy returns last: anhedonia and treatment resistance in depressed adolescents. J Am Acad Child Adolesc Psychiatry.

[10] Blank TS, Meyer BM, Wieser MK, Rabl U, Schögl P, Pezawas L (2022). Brain morphometry and connectivity differs between adolescent- and adult-onset major depressive disorder. Depress Anxiety.

[11] Gabbay V, Johnson AR, Alonso CM, Evans LK, Babb JS, Klein RG (2015). Anhedonia, but not irritability, is associated with illness severity outcomes in adolescent major depression. J Child Adolesc Psychopharmacol.

[12] McMakin DL, Olino TM, Porta G, Dietz LJ, Emslie G, Clarke G (2012). Anhedonia predicts poorer recovery among youth with selective serotonin reuptake inhibitor treatment-resistant depression. J Am Acad Child Adolesc Psychiatry.

[13] Zhang B, Lin P, Shi H, Öngür D, Auerbach RP, Wang X (2016). Mapping anhedonia-specific dysfunction in a transdiagnostic approach: an ALE meta-analysis. Brain Imaging Behav.

[14] Ng TH, Alloy LB, Smith DV (2019). Meta-analysis of reward processing in major depressive disorder reveals distinct abnormalities within the reward circuit. Transl Psychiatry.

[15] Arrondo G, Segarra N, Metastasio A, Ziauddeen H, Spencer J, Reinders NR (2015). Reduction in ventral striatal activity when anticipating a reward in depression and schizophrenia: a replicated cross-diagnostic finding. Front Psychol.

[16] Peciña M, Sikora M, Avery ET, Heffernan J, Peciña S, Mickey BJ (2017). Striatal dopamine D2/3 receptor-mediated neurotransmission in major depression: implications for anhedonia, anxiety and treatment response. Eur Neuropsychopharmacol.

[17] Pan PM, Sato JR, PaillèreMartinot ML, Martinot JL, Artiges E, Penttilä J (2022). Longitudinal trajectory of the link between ventral striatum and depression in adolescence. Am J Psychiatry.

[18] Stringaris A, Vidal-RibasBelil P, Artiges E, Lemaitre H, Gollier-Briant F, Wolke S (2015). The brain’s response to reward anticipation and depression in adolescence: dimensionality, specificity, and longitudinal predictions in a community-based sample. Am J Psychiatry.

[19] Ishida T, Dierks T, Strik W, Morishima Y (2020). Converging resting state networks unravels potential remote effects of transcranial magnetic stimulation for major depression. Front Psychiatry.

[20] Wang X, He K, Chen T, Shi B, Yang J, Geng W (2021). Therapeutic efficacy of connectivity-directed transcranial magnetic stimulation on anticipatory anhedonia. Depress Anxiety.

[21] Zhong G, Yang Z, Jiang T (2021). Precise modulation strategies for transcranial magnetic stimulation: advances and future directions. Neurosci Bull.

[22] Cole EJ, Phillips AL, Bentzley BS, Stimpson KH, Nejad R, Barmak F (2022). Stanford neuromodulation therapy (SNT): a double-blind randomized controlled trial. Am J Psychiatry.

[23] Cole EJ, Stimpson KH, Bentzley BS, Gulser M, Cherian K, Tischler C (2020). Stanford accelerated intelligent neuromodulation therapy for treatment-resistant depression. Am J Psychiatry.

[24] Ge R, Humaira A, Gregory E, Alamian G, MacMillan EL, Barlow L (2022). Predictive value of acute neuroplastic response to rTMS in treatment outcome in depression: a concurrent TMS-fMRI trial. Am J Psychiatry.

[25] Diana M, Raij T, Melis M, Nummenmaa A, Leggio L, Bonci A (2017). Rehabilitating the addicted brain with transcranial magnetic stimulation. Nat Rev Neurosci.

[26] Kinney KR, Hanlon CA (2022). Changing cerebral blood flow, glucose metabolism, and dopamine binding through transcranial magnetic stimulation: a systematic review of transcranial magnetic stimulation-positron emission tomography literature. Pharmacol Rev.

[27] Chan AW, Tetzlaff JM, Gøtzsche PC, Altman DG, Mann H, Berlin JA (2013). SPIRIT 2013 explanation and elaboration: guidance for protocols of clinical trials. BMJ (Clinical research ed).

[28] Costi S, Morris LS, Kirkwood KA, Hoch M, Corniquel M, Vo-Le B (2021). Impact of the KCNQ2/3 channel opener ezogabine on reward circuit activity and clinical symptoms in depression: results from a randomized controlled trial. Am J Psychiatry.

[29] Hamilton M (1960). A rating scale for depression. J Neurol Neurosurg Psychiatry.

[30] Montgomery SA, Asberg M (1979). A new depression scale designed to be sensitive to change. Br J Psychiatry.

[31] Gard DE, Gard MG, Kring AM, John OP (2006). Anticipatory and consummatory components of the experience of pleasure: a scale development study. J Res Pers.

[32] Fang S, Huang X, Zhang P, He J, Luo X, Zhang J (2021). Factor structure and sex invariance of the temporal experience of pleasure scale (TEPS) in Chinese university students and clinical population. BMC Psychiatry.

[33] Beck AT, Kovacs M, Weissman A (1979). Assessment of suicidal intention: the Scale for Suicide Ideation. J Consult Clin Psychol.

[34] Zhang J, Brown GK (2007). Psychometric properties of the scale for suicide ideation in China. Arch Suicide Res.

[35] Chung KF, Kan KK, Yeung WF (2011). Assessing insomnia in adolescents: comparison of insomnia severity index, Athens insomnia scale and sleep quality index. Sleep Med.

[36] Bastien CH, Vallières A, Morin CM (2001). Validation of the Insomnia Severity Index as an outcome measure for insomnia research. Sleep Med.

[37] Guy W (1976). ECDEU assessment manual for psychopharmacology: US Department of Health, Education, and Welfare, Public Health Service.

[38] Templin C, Hänggi J, Klein C, Topka MS, Hiestand T, Levinson RA (2019). Altered limbic and autonomic processing supports brain-heart axis in Takotsubo syndrome. Eur Heart J.

[39] Harrison JE, Barry H, Baune BT, Best MW, Bowie CR, Cha DS (2018). Stability, reliability, and validity of the THINC-it screening tool for cognitive impairment in depression: A psychometric exploration in healthy volunteers. Int J Methods Psychiatr Res.

[40] Xuemin Z (2022). Efficacy and safety of robot-assisted repetitive transcranial magnetic stimulation combined with sertraline in adolescent depressive disorder [master]: Department of Psychiatry, The First Hospital of Shanxi Medical University, Taiyuan, China.

[41] Pajula J, Tohka J (2016). How many is enough? effect of sample size in inter-subject correlation analysis of fMRI. Comput Intell Neurosci.

[42] Fukuda AM, Kang JWD, Gobin AP, Tirrell E, Kokdere F, Carpenter LL (2021). Effects of transcranial magnetic stimulation on anhedonia in treatment resistant major depressive disorder. Brain Behav.

[43] Auerbach RP, Pagliaccio D, Hubbard NA, Frosch I, Kremens R, Cosby E (2022). Reward-related neural circuitry in depressed and anxious adolescents: a human connectome project. J Am Acad Child Adolesc Psychiatry.

[44] Russo SJ, Nestler EJ (2013). The brain reward circuitry in mood disorders. Nat Rev Neurosci.

[45] Sharma A, Wolf DH, Ciric R, Kable JW, Moore TM, Vandekar SN (2017). Common dimensional reward deficits across mood and psychotic disorders: a connectome-wide association study. Am J Psychiatry.

[46] Whitton AE, Pizzagalli DA, Pizzagalli DA (2022). Anhedonia in depression and bipolar disorder. Anhedonia: Preclinical, Translational, and Clinical Integration.

[47] Eckstrand KL, Forbes EE, Bertocci MA, Chase HW, Greenberg T, Lockovich J (2019). Anhedonia reduction and the association between left ventral striatal reward response and 6-month improvement in life satisfaction among young adults. JAMA Psychiat.

[48] Gotlib IH, Hamilton JP, Cooney RE, Singh MK, Henry ML, Joormann J (2010). Neural processing of reward and loss in girls at risk for major depression. Arch Gen Psychiatry.

[49] Tang A, Harrewijn A, Benson B, Haller SP, Guyer AE, Perez-Edgar KE (2022). Striatal activity to reward anticipation as a moderator of the association between early behavioral inhibition and changes in anxiety and depressive symptoms from adolescence to adulthood. JAMA Psychiat.

