# Supplementary material for: Active versus sham DLPFC-NAc rTMS for depressed adolescents with anhedonia using resting-state functional magnetic resonance imaging (fMRI): a study protocol for a randomized placebo-controlled trial
Source: Trials. 2024 Jan 13;25:44. doi: 10.1186/s13063-023-07814-y (PMC10787505; doi:10.1186/s13063-023-07814-y)
Supplement: Supplementary file 1 — Additional file 1. [file 13063_2023_7814_MOESM1_ESM.zip › 5-11-V4.0-Informed Consent Form (caregivers)_ESM.docx]

Version 4.0 Date:11-May-2023

Informed Consent Form (caregivers)

Section1 Inform

Dear caregivers:

We invite your son or daughter to participate in a clinical study that has been approved by the Ethics Committee of Xijing Hospital. Please read the following content as carefully as possible before making decision, which will help you to understand this study, why it will be conducted, the procedure and duration of the study, and the benefits, risks, and discomfort that may be brought to your child if participating. You can also discuss it with your family and friends if you are willing to, and carefully decide whether to participate in this study. Feel free to raise questions and ask him or her to explain what you do not understand, when discussing with the researcher about the informed consent.

**1.Title of the study**

The Safety and efficacy of real versus sham DLPFC-NAc rTMS for depressive adolescent with anhedonia using resting-state functional magnetic resonance imaging(fMRI).

**2.Study profile**

Major depressive disorder (MDD) is common mental disorder, with anhedonia as one of its core symptoms. The traditional treatment for MDD is pharmacotherapy, which however seems ineffective for anhedonia. Repetitive transcranial magnetic stimulation (rTMS) is a safe and noninvasive neuromodulation technology for treating adult patients with MDD and has been approved by the US Food and Drug Administration; while its efficacy for adolescent patients with MDD is still unclear. According to previous studies, the efficacy of rTMS is associated with the precision of the stimulation target. Accordingly, adopting individualized precise targets is significant for improving the treatment efficacy. Therefore, this study aims to explore the efficacy and potential mechanism of the individualized targeting DLPFC-NAc rTMS with fMRI guide intelligent neural navigation system for treating anhedonia in adolescent patients with MDD, which is assumed to provide a new treatment choice and to have better curative effect.

**3.Study content and method**

This is a study initiated by the Psychosomatic Department of the Xijing Hospital in Shaanxi Province, China. A total of 88 adolescent patients with MDD will be recruited.

After knowing the possible risks and benefits of the study, if you and your child volunteer to participate and sign the informed consent and your child is assessed possibly eligible for the study, researchers will then collect demographic information and conduct physical examinations and neuropsychological testing on your child. Specifically, researchers will have a comprehensive knowledge of the anamnesis, family history, past suicidal situation and etc. of your child; and complete clinical and cognitive measurement scales. If your child meets the inclusion criteria; he or she will first have brain structural MRI and facial MRI for making 3D printed face tracer, and be randomly assigned to the experimental group or the control group to receive rTMS treatment with navigation robot. Your child has a 50% chance of being placed in the experimental group (active rTMS combined with antidepressants/drug therapy) and a 50% chance of being placed in the control group (pseudo rTMS combined with antidepressants). This study consists of a 15-day treatment phase (one 30-minute session of rTMS per day for 15 consecutive days) and an 8-week follow-up phase where clinical assessment scales will be implemented at pre-treatment, 7 days after treatment, 15 days after treatment, 4 weeks after treatment, and 8 weeks after treatment. Resting-state fMRI scans will be conducted before and after the rTMS treatment. It is important to note that antidepressant is required to be taken during the screening phase, please supervise your child to regularly take the medicine at the prescribed dose every morning.

**4.Study process and term.**

The study will last for 9 weeks, including pre-treatment physical examination, data collection, rTMS treatment, and post-treatment clinical follow-up and evaluation.

**5. Inclusion and Exclusion criteria**

It is an experimental study. Subjects should meet the following criteria to be enrolled in this study:

(1)13 to 18 years of age (including 13 but not 18). Either gender.

(2) Current diagnosis of MDD and in a MDE, according to the 5th edition of Diagnostic and Statistical Manual of Mental Disorders.

(3) The total score of HAMD-17 equals or exceeds 17 at both screening and baseline visits

(4) The total score of SHAPS equals or exceeds 20 at both screening and baseline visits.

(5) Having not taken anti-depressants for two weeks and more before screening.

(6) In good general health, as ascertained by the medical history.

(7) Being able to fully understand and strictly follow the clinical rTMS treatment protocol, and sign the informed consent.

In the meanwhile, subjects who meet any of the following criteria will be excluded from the study:

(1) Current diagnosis of a substance use disorder, with the exception of nicotine and caffeine dependence.

(2) Current diagnosis of mental disorders other than dysthymic disorder, generalized anxiety disorder, social anxiety disorder, panic disorder, agoraphobia, or specific phobia (unless one of these is clinically unstable, and/or the focus of the participant’s treatment for the past six months or more).

(3) History of schizophrenia or schizoaffective disorders, or any history of psychotic symptoms in the current or previous depressive episodes.

(4) Any other mental disorders, personality disorders, intellectual disability, which at screening is clinically predominant to their MDD.

(5) Having a clinically significant abnormality on the screening examination that might affect safety, study participation, or confound interpretation of study results.

(6) Any current or past history of any physical condition which in the investigator’s opinion might put the subject at risk or interfere with study results interpretation.

(7) Participation in any clinical trial with an investigational drug or device within the past month or concurrent to study participation.

(8) History of electronic instrument or metal in the head or skull.

(9) History of epilepsy.

(10) History of cardiovascular disease or cardiac event.

(11) History of obsessive-compulsive disorder.

(12) History of autism spectrum disorder.

(13) History of rTMS exposure.

(14) taking any antidepressants in the recent two weeks before screening.

(15) Other situations judged unsuitable as the studied subject by the researchers

Researchers have the right to terminate your child's participation in this study under the following circumstances

(1) Your child does not meet the selection criteria.

(2) The researchers believe that it is more beneficial to choose other treatments for the sake of your child's health.

(3) Your child is not suitable to continue to participate in this study because of other factors.

(4) laws and regulations factors.

(5) The study was suspended as a whole.

**6.Potential benefits**

(1) Subject benefits:

The direct medical benefit of this study is likely to improve your child's symptom during this period. Your child will receive professional treatment and close follow-up from doctors to guide your child's recovery.

(2) Expected social benefits:

The clinical data provided by your child will probably help more patients like your child and add more useful information for the medical development.

**7.Possible risk and discomfort**

MRI is known to have very little harm or side effects on the human body. Unlike X-rays and CT examinations, MRI has no radiation to the human body, and the greatest risk is that a metal attracted by the magnetic field can hit the body during its flight.

To reduce this risk, we require all participants and inspectors to remove all metal objects from their bodies before entering the magnetic room.

Please let us know in time if your child is afraid of the enclosed space, despite MRI scan will not bring discomfort itself. The scan lasts about 30 minutes. There will be some noise during the scan, especially during the starting few minutes in order to reconstruct individualized brain information better. We will provide earbuds for your child later to decrease the noise maximally.

It's important to keep stationary during the scan, The fillings used to fix the head generally feel comfortable, but please tell the researchers if your child feels uncomfortable. You and child have the right to terminate the study at any time without any reason.

The main risk and discomfort of neuropsychological testing is tiredness. Yourchild can request for rest anytime in testing.

RTMS is a noninvasive and secure neuromodulation intervention approved in many countries for depressive disorder, obsessive-compulsive disorder, neurogenic pain, and is widely used in insomnia, addiction, Alzheimer, Parkinson, rehabilitative period of stroke. Except for people who are equipped with magnetic, electronic, metal, or mechanical devices (e.g., pacemakers, metal dentures), the known harm and side-effects on the human body are very little, and the most common ones are mild headache and dizziness, which will be naturally relieved without special treatment. The incidence of epilepsy induced by rTMS is extremely low, with only 24 seizures reported in all rTMS research, less than 0.01%. This is actually closely related to the stimulus parameters (such as frequency, intensity, time, interval) and the type of coil. The stimulation parameters and coils use in this study are less likely to induce epilepsy. Epileptic seizures induced by rTMS are self-limiting and temporary, with no long-term effects.

If your child has any of the above discomfort or other accidents, please tell your child's research doctor immediately, and we will take care of it in a conscientious and timely manner. If any serious adverse event such as epilepsy occurs, the study will be terminated immediately, and hospitalization or adjustment of treatment will be coordinated.

**8. Treatment and economic compensation for experiment-related injuries**

In the event of any injury related to this study, your child can receive free treatment from the Psychosomatic Department of Xijing Hospital, and XIjing Hospital will make compensation in accordance with relevant laws and regulations.

**9. The confidentiality of medical records**

The research physician has the responsibility to protect your child's health, dignity, self-determination and privacy, and to keep your child's personal information confidential. All information in this study is very important, and it will be only used for research purpose, without any commercial or other use. The child's information is entered into the database in the form of code, which does not contain any personal identification information. Researchers will guarantee the privacy and information security of you and your family. Furthermore, the child's information may be monitored by the relevant departments (Ethics Committee, Food and Drug Administration), but its contents will not be disclosed to the public.

**10. Free diagnosis and treatment programs that may be available during the trial**

After you and your child sign the informed consent form, our scale evaluation, neurocognitive function test, MRI, 3D printing mask of navigation robot and rTMS therapy are all provided to your child free of charge by the Psychosomatic Department of Xijing Hospital.

The antidepressants used in this study are sertraline hydrochloride tablets which should be paid by yourself.

**11. The rights of subjects**

You and your child can ask any questions about this study at any time and get answers accordingly. The doctor will notify you and your child the new important information timely, which may affect your and your child's willingness to participate in the study.

Participate in this study on a voluntary basis. You and child can ask to withdraw at any time for any reason. It will not affect your and your family's right to health care or other services, if you do not participate in. Please contact the researcher if you have any questions during the study.

If the subjects have any questions in the study, please contact your research doctor, Runxin Lv, her phone number is 18049016906. The trial scheme was approved by the Hospital Ethics Committee, and the subjects can complain directly to the Committee if there is any violation of the research scheme during the experiment. The phone number is 029-84771784, and email is EC84771794@163.com.

Section2 consent

1.I have read the informed consent form carefully; researchers have given me detailed explanations and answered my related questions. I am fully aware of the above contents and agree to participate in the study.

Legal representative of subject signature： Date：

2. My researchers and I have fully explained to the subjects the purpose, procedure of this clinical trial, the possible risks and potential benefits of the subject’s participation in the trial, and satisfactorily answered all the relevant questions of the subjects.

the principal or the designated researcher signature: Date：
