# Supplementary material for: Active versus sham DLPFC-NAc rTMS for depressed adolescents with anhedonia using resting-state functional magnetic resonance imaging (fMRI): a study protocol for a randomized placebo-controlled trial
Source: Trials. 2024 Jan 13;25:44. doi: 10.1186/s13063-023-07814-y (PMC10787505; doi:10.1186/s13063-023-07814-y)
Supplement: Supplementary file 1 — Additional file 1. [file 13063_2023_7814_MOESM1_ESM.zip › 5-11-V4.0-Informed Consent Form (teenager)_ESM.docx]

Version 4.0 Date:11-May-2023

Informed Consent Form (teenager)

Section1 Inform

Dear patients:

How are you? Recently, you may have some discomfort, which has affected your study and life, so you come to see a doctor. The doctor thinks you may have adolescent depressive disorder and match the inclusion criteria for our study. We therefore invite you to participate in a clinical study held by the psychosomatic department and approved by the Ethics Committee of Xijing Hospital. It is a study on the safety and efficacy of the real versus sham DLPFC-NAc rTMS for depressive adolescent with anhedonia using resting-state functional magnetic resonance imaging(fMRI).

The informed consent form is to introduce the study objective, what you should do in the course of research, your potential benefits and possible discomforts and risks. Please read it carefully before deciding whether to participate. You can have a conversation with your parents (or other guardians) before making your decision, and your parents (or other guardians) will also have an informed consent form to read and sign.

**1. Why is this study carried out?**

Your emotional state has a great impact on your study and life, and similar situations happen to many other people of the same age. We will explore the effect of rTMS in the treatment of adolescent depressive disorder with anhedonia. The therapeutic effect of rTMS in adult patients with anhedonia has been proved, but it remains a potential treatment choice that have not yet been identified for teenagers. In addition, this study will also explore the relevant brain neuroimaging mechanism of rTMS through MRI.

**2. How many subjects will participate in the study?**

The study will recruit 88 adolescents aged between13-18 years old like you(including 13, excluding 18).

**3. How is the study conducted?**

**3.1 Study methodology**

After knowing the possible risks and benefits of the study, if you and your parents volunteer to participate and sign the informed consent and you are assessed possibly eligible for the study, researchers will then collect demographic information and conduct physical examinations and neuropsychological testing on you. Specifically, researchers will have a comprehensive knowledge of the anamnesis, family history, past suicidal situation and etc. of you; and complete clinical and cognitive measurement scales. If you meet the inclusion criteria; you will first have brain structural MRI and facial MRI for making 3D printed face tracer, and be randomly assigned to the experimental group or the control group to receive rTMS treatment with navigation robot. The method of grouping is similar to flipping a coin. You have a 50% chance of being placed in the experimental group (active rTMS combined with antidepressants/drug therapy) and a 50% chance of being placed in the control group (pseudo rTMS combined with antidepressants).

**3.2 The treatment and follow-up schedule**

This study consists of a 15-day treatment phase (one 30-minute session of rTMS per day for 15 consecutive days) and an 8-week follow-up phase which is to observe your treatment effectivity where clinical assessment scales will be implemented at pre-treatment, 7 days after treatment, 15 days after treatment, 4 weeks after treatment, and 8 weeks after treatment. Resting-state fMRI scans will be conducted before and after the rTMS treatment. It is important to note that antidepressant is required to be taken during the screening phase, please regularly take the medicine at the prescribed dose after breakfast every morning.

**4.** **What do you need to do?**

If you participate in this study, you need to:

(1) Tell your doctor what is ailing for you or what disease you've had before;

(2) Receive standardized examination and treatment according to the plan made by the doctor;

(3) Only with the permission from the doctor can you receive other medicines or treatment, and the doctor will keep a record;

(4) Tell the doctor about any discomfort you have during the study;

(5) Follow the doctor's instructions;

(6) If there is anything unclear, ask your doctor or parents at any time.

**5. What benefits are you likely to get?**

(1) Subject benefits:

The direct medical benefit of this study is likely to improve your symptom during this period. Your will receive professional treatment and close follow-up from doctors to guide your recovery.

(2) Expected social benefits:

The clinical data that you provided will probably help more patients like you and add more useful information for the medical development.

**6.** **What discomfort may you feel?**

MRI is known to have very little harm or side effects on the human body. Unlike X-rays and CT examinations, MRI has no radiation to the human body, and the greatest risk is that a metal attracted by the magnetic field can hit the body during its flight. To reduce this risk, we require all participants and inspectors to remove all metal objects from their bodies before entering the magnetic room.

Please let us know in time if you are afraid of the enclosed space, despite MRI scan will not bring discomfort itself. The scan lasts about 30 minutes. There will be some noise during the scan, especially during the starting few minutes in order to reconstruct individualized brain information better. We will provide earbuds for you later to decrease the noise maximally.

It's important to keep stationary during the scan, The fillings used to fix the head generally feel comfortable, but please tell the researchers if you feel uncomfortable. You have the right to terminate the study at any time without any reason.

The main risk and discomfort of neuropsychological testing is tiredness. You can request for rest anytime in testing.

RTMS is a noninvasive and secure neuromodulation intervention approved in many countries for depressive disorder, obsessive-compulsive disorder, neurogenic pain, and is widely used in insomnia, addiction, Alzheimer, Parkinson, rehabilitative period of stroke. Except for people who are equipped with magnetic, electronic, metal, or mechanical devices (e.g., pacemakers, metal dentures), the known harm and side-effects on the human body are very little, and the most common ones are mild headache and dizziness, which will be naturally relieved without special treatment. The incidence of epilepsy induced by rTMS is extremely low, with only 24 seizures reported in all rTMS research, less than 0.01%. This is actually closely related to the stimulus parameters (such as frequency, intensity, time, interval) and the type of coil. The stimulation parameters and coils use in this study are less likely to induce epilepsy. Epileptic seizures induced by rTMS are self-limiting and temporary, with no long-term effects.

If you have any of the above discomfort or other accidents, please tell your research doctor immediately, and we will take care of it in a conscientious and timely manner. If any serious adverse event such as epilepsy occurs, the study will be terminated immediately, and hospitalization or adjustment of treatment will be coordinated.

**7.** **What if there is an incidence?**

In the event of any injury related to this study, you can receive free treatment from the Psychosomatic Department of Xijing Hospital, and XIjing Hospital will make compensation in accordance with relevant laws and regulations.

**8. Will my information be disclosed ?**

All information about you and your family is confidential. The research physician has the responsibility to protect your health, dignity, self-determination and privacy, and to keep your personal information confidential. All information in this study is very important, and it will be only used for research purpose, without any commercial or other use. The information is entered into the database in the form of code, which does not contain any personal identification information. Researchers will guarantee the privacy and information security of you and your family. Furthermore, your information may be monitored by the relevant departments (Ethics Committee, Food and Drug Administration), but its contents will not be disclosed to the public.

**9. What free diagnosis and treatment programs can I get during the trial?**

After you sign the informed consent form, our scale evaluation, neurocognitive function test, MRI, 3D printing mask of navigation robot and rTMS therapy are all provided to you free of charge by the Psychosomatic Department of Xijing Hospital.

The antidepressants used in this study are sertraline hydrochloride tablets, which should be paid by yourself.

**10. What rights do I have?**

You can ask any questions about this study at any time and get answers accordingly. The doctor will notify you the new important information timely, which may affect your willingness to participate in the study.

Participate in this study on a voluntary basis. You can ask to withdraw at any time for any reason. It will not affect your right to health care or other services, if you do not participate in. Please contact the researcher if you have any questions during the study.

If the subjects have any questions in the study, please contact your research doctor, Runxin Lv, her phone number is 18049016906. The trial protocol was approved by the Hospital Ethics Committee, and the subjects can complain directly to the Committee if there is any violation of the research scheme during the experiment. The phone number is 029-84771784, and email is EC84771794@163.com.

Section2 consent

1.I have read the informed consent form carefully; researchers have given me detailed explanations and answered my related questions. I am fully aware of the above contents and agree to participate in the study.

Subject signature： Date：

Legal representative of subject signature (when necessary) Date：

2. My researchers and I have fully explained to the subjects the purpose, procedure of this clinical trial, the possible risks and potential benefits of the subject’s participation in the trial, and satisfactorily answered all the relevant questions of the subjects.

the principal or the designated researcher signature: Date：
